# News and Events of the Week

**Published:** 1909-10-02

**Authors:** 


### News and Events of the Week.

Dr. G. Munro Smith, M.R.C.S., has been appointed consulting surgeon to the Bristol Royal Infirmary.

Professor J. Michell Clarke, M.D., has been appointed Pro-Vice-Chancellor of the University of Bristol.

Mr. H. F. Mole, F.R.C.S., has been appointed surgeon, and Mr. C. F. Walters, F.R.C.S., assistant-surgeon, to the Bristol Royal Infirmary.

The Bombay University has decided to abolish the medical degree of L.M. and S. (Licentiate in Medicine and Surgery) and substitute one equivalent to the M.B. in England. The Government has been moved to sanction the recommendation.

A labourer, thirty years of age, died after a sudden illness at Stolzenhagen, near Stettin. A bacteriological examination showed definitely that it was a case of Asiatic cholera. This is the first case of cholera that has been reported from Germany.

"Wonderer" writes to the *British and Colonial Druggist* as follows: "I had a 'scrip' in last night which might interest you. It was written as follows: ℞ Ac. boracis, gr. v.; sod. sulphat., gr. 1; aq. ad ℥j. Sj. Drip a drop into the eyes nightly for three weeks. Evidently *boracis* is used instead of *borici*, and sod. sulph. I believe was meant for zinci sulph. The prescription was sent with a letter from 'Carmelite House,' apparently in answer to a query sent to the medical column of one of their papers."

The Home Secretary has signified to the Council of the Royal College of Surgeons his decision to approve the by-laws regarding the admission of women to the examinations for the diplomas of the College, and has further expressed his willingness to sign the formal document to be submitted after the next meeting of the Council of the College on October 14. It is understood, however, that meanwhile it will be possible to complete the necessary formalities in time for women to enter for the examinations of the Royal College to be held in January next.

Considerable progress has been made with the organisation of the county sections of the new Territorial branch of the St. John Ambulance Association, the special object of which is to co-operate with the County Associations and the British Red Cross Society in the provision of recruits for the voluntary aid detachments being organised by the latter body. Among those who have already accepted the office of presidents of county sections are Princess Christian (Berks), the Duke and Duchess of Devonshire (Derbyshire), the Duke and Duchess of Portland (Notts), the Duke and Duchess of Rutland (Leicester), Lord and Lady Bristol (Suffolk).

Dr. Brown, of the Pretoria Lunatic Asylum staff, has received the Royal Humane Society's medal. In February last Dr. Brown plunged into a deep quarry pool, the bottom of which was covered with barbed wire, in an attempt to save a man, Walters, who had gone in to bathe and who got out of his depth. The rescue involved a good deal of peril, but Dr. Brown persevered until he recovered the body, but the rescue was too late to restore life. Mr. Halling, an assistant at the Asylum, also rendered plucky assistance. The circumstances were brought to the notice of the Society, with the result that a medal was awarded to Dr. Brown and a testimonial to Mr. Halling.

Dr. Crosby Johnson has resigned the post of Honorary Physician to the Pendleton Branch Dispensary.

A dental school clinic, supported by a levy of 20 pfennig per annum on each pupil attending the city schools, will be opened at Hamburg this month.

Dr. R. J. Collie, J.P., medical superintendent of the first-aid, home nursing, health, and infant-care classes connected with the Education Committee of the London County Council, has resigned his position.

Dr. Frederic W. Hewitt, M.V.O., will read a paper on "The need for legislation in regard to anæsthetics and the lines upon which it should take place" before the Medical Society of London, on Monday, October 25.

Though only subscribers will be allowed to vote at the general meeting of the Church and Medical Union at Church House on October 11, the public will be welcome to hear the speeches, and Mr. Geoffrey Rhodes, the secretary, will be glad to send cards of admittance on receipt of stamped envelope sent to him at Dryden House, 43 Gerrard Street.

We have to record the death on September 8 of Dr. Thomas Bridgwater, who was President of the Council of the British Medical Association from 1887 to 1889. Dr. Bridgwater, who entered the profession in 1847, and graduated M.B.Lond. in 1852, practised for many years at Harrow. He retired some time ago to Hoote Hall, Uckfield, and it was there that his death took place.

Mr. J. Bland-Sutton, F.R.C.S., has made a donation of £1,000 towards the fund which is being raised for the purchase of an athletic ground for the Middlesex Hospital Medical School. By Mr. Bland-Sutton's generous action the amount required has been reduced to £1,500, and it is hoped that this sum will be obtained from an appeal which is being made to former students and others interested in the school.

The death is announced of Dr. E. R. St. Clair Corbin, at the age of 51 years. Dr. Corbin was a student of University College Hospital Medical School, and graduated M.B. London in 1886, gaining honours in forensic medicine and Materia Medica. The following year he took the M.R.C.S. England diploma, and was house surgeon at University College Hospital. Later on he settled in general practice at Beckenham.

At the monthly meeting of the Board of Management of the Salford Royal Hospital, the following resident medical officers were appointed for the six months from the 1st October:—Resident Surgical Officer, E. A. Walker, M.B., Ch.B. (Edin.); House Physician, Arthur C. Brown, M.B., Ch.B. (St. Andrews); House Surgeon, C. H. Broomhead, M.B., Ch.B. (Vict.), M.B., B.S. (Lond.); Junior House Surgeon, A. Gordon McLeod, M.B., Ch.B. (Edin.).

The following gentlemen have been elected to entrance scholarships at St. Mary's Hospital Medical School:—University Scholarships of 50 guineas each: Horatio Thomas and T. H. Phillips, both of University College, Cardiff. Open Scholarships in Natural Science: £145, R. W. Davies, King Edward's School, Birmingham; £50, F. W. MacAlevey, Mount St. Mary's College, Chesterfield; £25, J. E. Cheesman, South-Western Polytechnic. Epsom College Scholarship: D. R. Alexander.

At Hansweert, a village in Zeeland, on the West Schelde, on which Flushing lies, two cases of cholera are officially stated to have occurred.

Dr. Arthur de Vine has been appointed medical officer for the fifth district of the Hertford Union, in place of Dr. W. A. D. King, resigned.

The will of Dr. Theodore Davis, of Haslemere, Walton-in-Gordano, Clevedon, surgeon to the Cottage Hospital, Clevedon, who died on August 11, aged 75, was proved at £17,213 gross and £16,178 net.

Dr. Thomas Crawford Hayes, of Clarges Street, Mayfair, W., Emeritus Professor of Medicine at King's College, who died on April 5, left £50,739 8s. 5d. gross, and £48,765 7s. 3d. net.

At the Conference on Social Questions, held, under the auspices of the League of Progressive Thought and Social Service, on Tuesday, October 12, a resolution will be moved by Dr. L. Haden Guest, seconded by Dr. Reg. Tribe, on the subject of "The Medical Treatment of School Children.".

A discussion will take place on "The Teaching of Therapeutics in the Hospital Wards" at the Therapeutical and Pharmacological Section of the Royal Society of Medicine, 20 Hanover Square, W., on Tuesday, October 5, at 4.30 p.m. The discussion will be opened by Sir Clifford Allbutt and Professor Osler, Dr. Harrington Sainsbury, Dr. Robert Hutchison, and Professor Dixon will take part in it.

At the Charing Cross Hospital Medical School the following entrance scholarships have been awarded: The Epsom Scholarship (60 guineas), to Mr. Duncan, W. Pailthorpe; the Livingstone Scholarship (75 guineas), to Mr. Francis J. Hallinan; the Huxley Scholarship (50 guineas), to Mr. David B. S. Jones. An entrance scholarship has also been awarded to Mr. Harold W. Williamson (30 guineas), a Universities Scholarship of 50 guineas to Mr. J. Milton Davies, and a Universities Exhibition of 20 guineas to Mr. Abel Evans, both of London University.

The next meeting of the Society for the Study of Inebriety will be held in the rooms of the Medical Society of London, 11 Chandos Street, Cavendish Square, W., on Tuesday, October 12, 1909, at 4 p.m. (afternoon meeting), when J. Milne Bramwell, M.D., C.M., author of "Hypnotism, Its History, Practice and Theory," will open a discussion on "Suggestion and its Rôle in the Treatment of Inebriety." Each member and associate is at liberty to introduce a visitor. Tea and coffee at 3.45. Council meeting at 3.30.

### HYGIENE IN A MODERN FACTORY.

Some time ago the Oxo Company started a scheme which had an interesting climax last week. The public, which had taken advantage of the scheme, was conducted not only over a large part of London but over the new model Oxo factory on the South side. There it had an opportunity of seeing what modern hygiene has done for the latter-day factor worker. The place looked more like a dairy than a factory, and in the houses erected for the employees, and in the heating and ventilation of the buildings were seen an extraordinary combination of scientific principle and practical result. The visitors made good use of the demonstration in practical hygiene they were fortunate enough to receive, and returned from their trip filled with admiration for the admirable manner in which foundation for the many popular stimulating beverages that may be made out of Oxo is manufactured.

Dr. Frederic Gardiner has been reappointed physician for diseases of the skin at the Edinburgh Royal Infirmary.

The Melbourne University Council has decided to appoint Dr. G. C. Mathieson, of St. Mary's Hospital, London, as demonstrator and assistant lecturer in physiology.

The death is announced of Mr. George Bulleid, M.R.C.S. Mr. Bulleid was born in 1821, and was a student at St. George's Hospital. He took his diploma in 1852.

The death is announced of Dr. Hugh T. Adams, of Jersey City, U.S.A., at his home as the result of a railroad accident. Dr. Adams was a native of Ireland and a graduate of Queen's College, Dublin, in 1869.

The death is announced of Mr. George Richard Turner Phillips, J.P., M.R.C.S., M.S.A. Mr. Phillips, who had retired from practice and lived at Folkestone, studied both at Guy's and at St. George's, taking his diploma in 1863.

The Pure Food Congress organised under the auspices of the Society of the White Cross of Geneva meets this year in Paris on October 17-28. There will be a special and wholly independent section devoted to drugs, essential oils, chemical products, medicinal waters, and ice. The general secretary for the United Kingdom is Mr. L. M. Douglas, 3 Lauder Road, Edinburgh.

The American Ambassador will preside at the opening of the winter session of the London School of Tropical Medicine on October 26. The subject of the address, by Professor Osler, will be "The Nation and the Tropics." In the evening Dr. Bagshawe, Director of the Sleeping-Sickness Bureau, will take the chair at the school dinner to be held at the Savoy.

In connection with the opening of the Session of the Faculty of Science and the Faculty of Medical Sciences at University College Professor Sir William Ramsay will lecture on "Radium Emanation; one of the Argon lines of Gases" on Monday, October 4, at 9 a.m., and Professor H. R. Kenwood on "What Hygiene Demands of School Teachers" on Wednesday, October 6, at 7 p.m.

### THE MEDICAL EXHIBITION.

The fifth London Medical Exhibition will be held in the New Hall of the Royal Horticultural Society, Vincent Square, Westminster, during the five days, Monday, Tuesday, Wednesday, Thursday, Friday, October 4th to 8th, 1909, from 12 noon to 7 p.m. daily. The endeavour of the promoters, who are the organisers of the very successful Chemists' Exhibitions held in London, to run their Medical Exhibitions on entirely new lines has been attended with the very best results. Indeed nearly every possible means is adopted to exclude the public, not the least effective being the entrance fee, 5s. There are about 7,000 medical men in the London district, and every inducement is made to get a large proportion of them to pay a visit. The last Exhibition was attended by over 3,000 London practitioners, in addition to a good number from the provinces and places abroad. A handsome reception room, where light refreshments will be provided, will be at the service of the profession. The management invites every medical man within a 20 mile radius of London. In order to keep the attendance select, and of a strictly professional character, no tickets for distribution will be supplied to exhibitors. Each doctor will have a secon ticket to admit himself and friend (lady or gentleman). The Exhibition will be advertised in the professional papers, and every medical man will have personal communications sent to him.

